# Proteomics data in support of the quantification of the changes of bovine milk proteins during mammary gland involution

**DOI:** 10.1016/j.dib.2016.05.013

**Published:** 2016-05-13

**Authors:** Irina Boggs, Brad Hine, Grant Smolenski, Kasper Hettinga, Lina Zhang, Thomas T. Wheeler

**Affiliations:** a Dairy Foods, AgResearch, Ruakura Research Centre, Private Bag 3123, Hamilton 3240, New Zealand; b CSIRO, Agriculture Flagship, Chiswick, Armidale, NSW 2350, Australia; c Wageningen University, Dairy Science and Technology, FQD Group, PO Box 17, 9, 6700AA Wageningen, The Netherlands

**Keywords:** Host defence, Dairy, Drying off, Proteomics

## Abstract

Here we provide data from three proteomics techniques; two-dimensional electrophoresis (2-DE) followed by identification of selected spots using PSD MALDI-TOF MS/MS, one-dimensional gel electrophoresis followed by LC-MS/MS analysis of gel slices (GeLC) and dimethyl isotopic labelling of tryptic peptides followed by Orbitrap MS/MS (DML), to quantify the changes in the repertoire of bovine milk proteins that occurs after drying off. We analysed skim milk and whey sampled at day 0 and either day 3 or day 8 after drying off. These analyses identified 45 spots by MALDI-TOF, 51 proteins by GeLC and 161 proteins by DML, for which the detailed data work-up is presented as three Excel files. The data supplied in this article supports the accompanying publication “Changes in the repertoire of bovine milk proteins during mammary involution” (Boggs et al., 2015) [1]. Data are available via ProteomeXchange with identifiers ProteomeXchange: PXD003110 and ProteomeXchange: PXD003011.

**Specifications Table**

**Table:** 

Subject area	Biology
More specific subject area	Mammary gland physiology
Type of data	Excel files
How data was acquired	Two-dimensional electrophoresis followed by PSD MALDI-TOF (LIFT) mass spectrometry of selected spots using a Bruker Ultraflex instrument. One-dimensional gel electrophoresis followed by LC-tandem MS of peptides excised from gel slices (GeLC) using a QStar instrument. Dimethyl labelling followed by LC tandem MS mass spectrometry (DML) using an Oribtrap intrument.
Data format	Raw
Experimental factors	Skim milk and whey from day 0 and day 3 or day 8 after drying off
Experimental features	Pooled skim milk and whey samples were separated using 2-DE and gel spots that were altered in abundance were excised, digested with trypsin and identified by MALDI-TOF. The pooled samples were also analysed by 1D electrophoresis, slices of each lane were subjected to trypsin digestion, and the extracted peptides were analysed by LC-MS/MS. Peptide abundance was estimated by peptide count and EMPAI value. The pooled samples were also digested, isotope labelled and subjected to LC-MS/MS. Isotope ratios were determined for each peptide.
Data source location	Waikato region of New Zealand
Data accessibility	Data is available within this article and through ProteomeXchange accession numbers ProteomeXchange: PXD003110 and ProteomeXchange: PXD003011.

## 1 Value of the data

• This data characterizes the physiological responses occurring in milk during mammary involution in dairy cows.
• Many host defence related proteins were increased in abundance after drying off.
• These data could be used for developing improved milking strategies to maximise production efficiency and yield of milk bioactives.

## 2 Data

Three Excel files are presented. File 1 contains the MALDI-TOF tandem MS data of the 2-DE gel spots which were successfully identified, File 2 contains the raw data excised from ProteinScape, the processed data, and a detailed description of the contents of each of the sheets, along with the workflow used. File 3 contains a single sheet containing collated data listing all the distinct proteins that were identified, along with the number of peptides detected, their isotope ratios, and biological function as determined by querying the GO database. A complete description of the data and methods is presented elsewhere [1].

## 3 Experimental design, materials and methods

### 3.1 Milk collection and sample preparation

Raw milk samples were collected from 12 pregnant pasture-fed Friesian-Holstein dairy cows at a single dairy farm. The cows were randomly split into two groups of six. The first group was sampled on day 0 (D03), the last day of milking, as well as day 3 after drying off (D3), and the second group was sampled on day 0 (D08) as well as day 8 after drying off (D8). Milk was obtained from each quarter for western blot analysis and an equal volume of milk from the four quarters of the six cows in each group was pooled for MS analysis. The milk samples were centrifuged at 1500x*g* for 20 min and the fat layer was removed before storage at −20 °C (skimmed milk). An aliquot of the skimmed milk was centrifuged at 100,000x*g* for 60 min at 30 °C to pellet the casein micelles, and the clear supernatant (whey) was removed and stored at −20 °C.

### 3.2 2-DE and protein identification

A 500 µg portion of the pooled samples from each of the conditions (D03, D3, D08, D8 for skimmed milk and whey) as determined by the Bradford protein assay (Bio-Rad) was subjected to 2-DE, each in duplicate, following a previously described method [2]. Image analysis was performed using the PDQuest v8.0.1 software package (Bio-Rad, Hercules, CA, USA), which produced integrated spot intensities (“volumes”) for each of the selected spots. PDQuest analysis revealed 91 spots for which the mean abundance between the replicate gels was altered at least two-fold between D0 and at least one of the time points (either D3 or D8). These 91 spots were excised from the stained gels and subjected to MALDI-TOF analysis on an Ultraflex mass spectrometer (Bruker, Billerica, MA, USA) with FlexControl 3.0 data acquisition software (Bruker). Peptide and fragment mass tolerances were set to 100 ppm for precursor ions and 0.6 Da for fragment ions. Tandem MS was performed on peptides using post source decay (LIFT-TOF). The data were subjected to peptide fragment fingerprinting to identify proteins using a cutoff value of 20 ppm. A MOWSE score producing a probability value of less than 0.05 that the match occurred by chance was considered to be a positive identification. MALDI-TOF analysis resulted in the positive identification of 45 spots. Identifications from single peptides were manually curated and considered valid only if (1) the peptide ion score was greater than 25, (2) a greater than 60% coverage of b and y ion series that were clearly above the noise levels, and (3) the MASCOT score was required to be significantly greater than the next best match to a different peptide. The data relating to each of the identified proteins, including their sequence, modifications, scores, and the peptide match error are presented in File 1.

### 3.3 GeLC analysis

The pooled samples were each subjected to one-dimensional SDS electrophoresis on separate lanes. Each lane was then cut into 10 equal slices and each slice was subjected to *in situ* trypsin digestion on the destained, reduced and alkylated gel pieces. The peptides were extracted from each slice and subjected separately to LC tandem MS using a QSTAR instrument (ABI) at a flow rate of 800 nl/min. Fragment ion peak lists were produced from the raw data using ProteinScape, and these were used to query MASCOT to identify the peptide and obtain EMPI values, which were then extracted into Excel. Search parameters for peptide and fragment mass tolerances were set to 200 ppm for MS and 0.6 Da for MS/MS, with allowance made for one missed tryptic cleavage. The peptide ion score threshold was set at 25. A significance threshold of less than 0.05 was selected and all searches were conducted using the peptide-decoy option selected within ProteinScape. This produced an average false discovery rate of 1.4%. The MASCOT search results are presented in File 2.

### 3.4 Quantitative proteomics using Dimethyl labelling (DML)

In order to complement the quantitative data from PDQuest analysis, we used the DML method to quantify the abundance of proteins in each condition. Changes in abundance of proteins between D03 (reference), D08, D3 and D8 in the pooled skimmed milk as well as pooled whey samples were determined using dimethyl labelling of peptides as previously described [3], [4]. The digested samples were labelled with methyl groups using either a light (D03) or a heavy (D3, D08, and D8) isotope formaldehyde reagent, the latter containing a deuterium isotope (Cortecnet, Paris, France) MS. Pairs of samples were mixed, and the peptides were resolved and detected by LC mass spectrometry using an LTQ Orbitrap mass spectrometer as detector and a flow rate of 500 nl/min. The MS/MS spectra were analysed and the H/L ratios were calculated using the Maxquant (v1.3.0.5) software package and the Andromeda search engine as previously described [4]. Peptide ratios were normalised based on setting the median of their logarithms at zero, which corrects for unequal protein loading, assuming that the majority of proteins show no differential regulation. Coefficients of variability of peptide ratios were also determined. Normalised H/L ratios were used for further statistical analysis. The function of the identified proteins was extracted from the UniprotKB database (http://www.uniprot.org/, release June, 2013).

The analysis resulted in the identification of 161 proteins. In 65 of those at least two peptides were detected in at least one of the pairwise comparisons and the mean of the log_2_ of the isotope ratio was either greater than 2 or less than −2, indicating at least a four-fold change in abundance. The full list of quantified proteins, number of identified peptides, ratios, sequence coverage and intensities of peptides as iBAQ numbers is presented in File 3.
